# How I approach: the transplant recipient with fever and pulmonary infiltrates

**DOI:** 10.3389/fped.2024.1273590

**Published:** 2024-02-19

**Authors:** Madan Kumar, Benjamin R. Hanisch

**Affiliations:** ^1^ Section of Pediatric Infectious Diseases, University of Chicago, Chicago, IL, United States; ^2^ Department of Pediatrics, School of Medicine and Health Sciences, Division of Infectious Diseases, Children’s National Hospital, The George Washington University, Washington, DC, United States

**Keywords:** pediatric, solid organ transplant (SOT), pulmonary infection, nodules, stem cell transplant (SCT), pulmonary infiltrate

## Abstract

Recipients of hematopoietic stem cell transplants and solid organ transplants frequently develop pulmonary infiltrates from both infectious and non-infectious etiologies. Differentiation and further characterization of microbiologic etiologies—viral, bacterial, and fungal—can be exceedingly challenging. Pediatric patients face unique challenges as confirmatory evaluations with bronchoscopy or lung biopsy may be limited. A generalizable approach to diagnosing and managing these conditions has not been well established. This paper aims to summarize our initial clinical approach while discussing the relative evidence informing our practices. A pediatric patient with characteristic infiltrates who has undergone HSCT is presented to facilitate the discussion. Generalizable approaches to similar patients are highlighted as appropriate while highlighting considerations based on clinical course and key risk factors.

## Introduction

Recipients of hematopoietic stem cell transplants (HSCTs) and solid organ transplants (SOTs) frequently develop pulmonary infiltrates from both infectious and non-infectious etiologies. Differentiation and further characterization of microbiologic etiologies, including viruses, bacteria, and fungi, can be exceedingly challenging. Pediatric patients face unique challenges due to limitations in evaluations, including sputum cultures, bronchoscopy, and lung biopsy. A generalizable algorithm for diagnosing and managing these conditions has not been well established. This paper aims to review the general framework and summarize our initial clinical approach while discussing the relative evidence informing our practices in a case-based manner.

**Case**: An 8-year-old girl with T-cell lymphoblastic lymphoma who underwent HSCT with a matched unrelated donor developed a fever and presented with progressive respiratory distress accompanied by a progressing oxygen requirement on day 41 after transplantation. Her conditioning regimen included total body irradiation and cyclophosphamide, while her prophylaxis included acyclovir, micafungin, and trimethoprim-sulfamethoxazole (initiated after engraftment). Her initial transplant clinical course was complicated by delayed engraftment and persistently low absolute neutrophil and lymphocyte counts; additionally, she had experienced prior adenovirus DNAemia, which was treated with cidofovir and has since resolved. Throughout the transplant process, weekly screening blood PCR tests for cytomegalovirus (CMV), Ebstein–Barr virus (EBV), and adenovirus were performed and yielded negative results.

***Question 1: What is the differential diagnosis for new pulmonary infections in a transplant recipient?***

The primary host risk factors for pulmonary infections in both HSCTs and solid organ transplants relate to the type of transplant, temporal proximity to transplantation, and the net state of immunosuppression, particularly in relation to treatment for rejection or graft-vs.–host disease (GvHD). In HSCT recipients, the risk profile for different pathogens varies depending on the timing of the transplant and the degree of immune reconstitution. Generally, the highest risk for severe infection occurs in the early stages of transplantation, which is associated with neutropenia and mucositis, and exists prior to functional T-cell recovery (1). Conditioning regimens and subsequent immunosuppression contribute to the risk of infection development. In general, myeloablative regimens (including total body irradiation) can lead to more severe neutropenia, mucositis, and organ toxicities and increase the overall risk of infection development. Risks are further increased in the presence of GvHD and with the increased use of immunosuppressive therapies as treatment (1).

For solid organ transplant recipients, infections shortly after transplantation are most common and can often be attributed to nosocomial or surgical infection (including typical bacterial infections), reactivation from induction therapy (viral hepatitis and *Herpesviridae* family viruses), or can be donor-derived (2). After the initial transplant period (approximately the first 30 days through the first 6 months), infections tend to be related to the activation of latent infections or opportunistic infections due to ongoing immunosuppression. Opportunistic infections caused by *Pneumocystis*, *Aspergillus*, CMV/EBV, and *Mycobacterium tuberculosis* may all be implicated in this timeframe. After the first 6–12 months, most patients with stable grafts and reduced immunosuppression will experience fewer opportunistic infections but continue to be at risk for community-acquired pneumonias, *Legionella*, respiratory viruses, and potentially CMV reactivation upon discontinuation of prophylaxis (2).

For all patients, an infection and exposure history should be included to obtain a differential diagnosis. The history should emphasize the patient's prior infectious history, including any history of multi-drug-resistant colonization or infection, travel to areas with endemic fungi, exposure to tuberculosis, contact with sick individuals, and any environmental disruptions that may stir up molds or endemic fungi.

**Figure 1:**
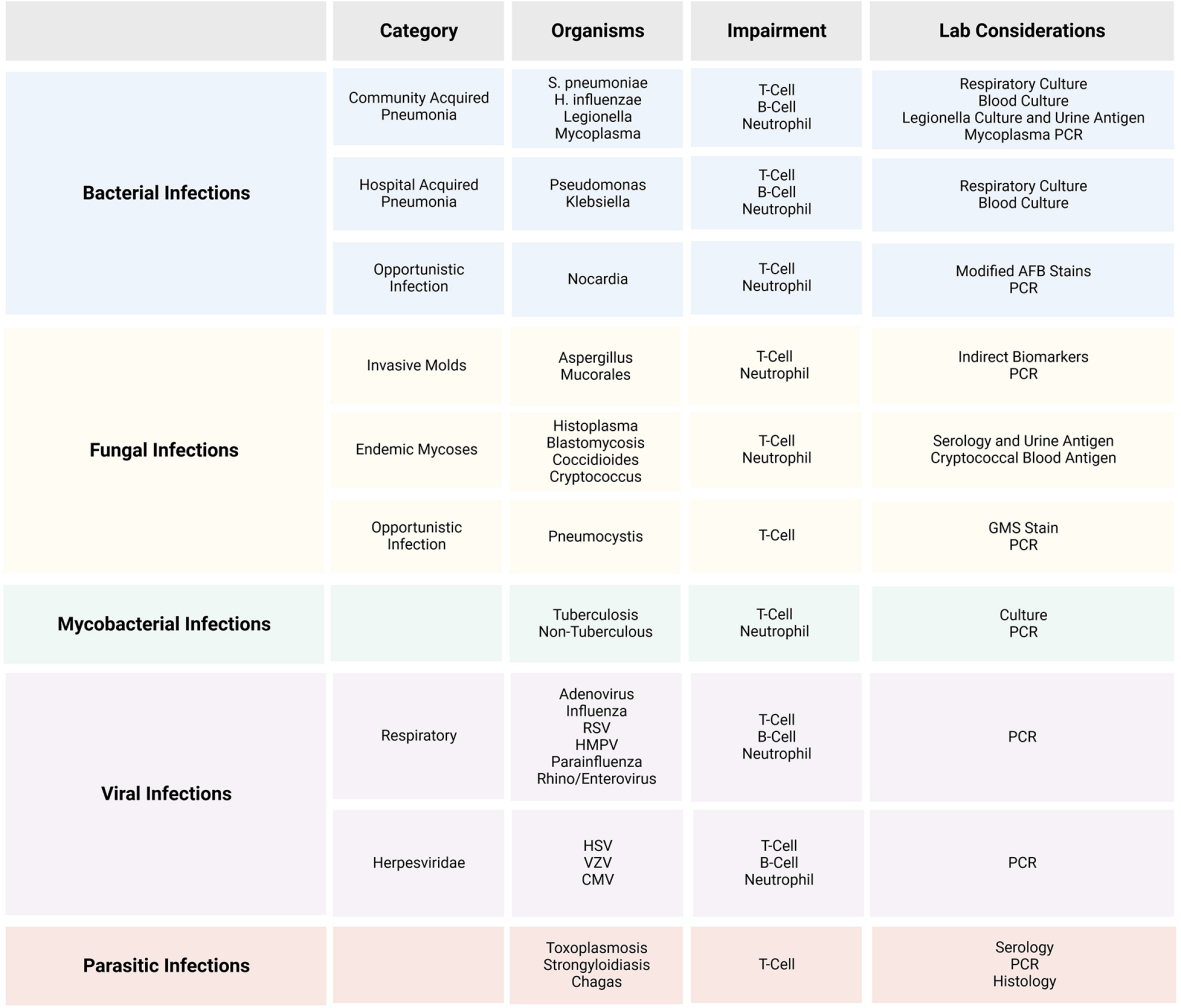
Common etiologic agents for invasive pulmonary disease in post-transplant recipients.

A broad array of potential etiologies should be considered in evaluating pulmonary infections in patients who underwent transplantation. Although bacterial pneumonias remain common, other etiologies including invasive molds such as *Aspergillus* and *Mucorales*, disseminated candidemia, endemic fungi such as *Histoplasma*, *Coccidioides*, and *Blastomyces* (depending upon the geography and travel history), and infections caused by *Nocardia*, mycobacteria, and *Pneumocystis jirovecii* (PCP) should all be considered (3). This study focuses on infectious etiologies, although non-infectious etiologies such as cryptogenic organizing pneumonia or cancer may present with similar initial symptoms (Figure 1).

Viral pneumonias cause substantial morbidity in SOT and HSCT recipients. A large multi-center study of pediatric HSCT recipients revealed that 16.6% acquired a respiratory viral infection during their hospitalization. Among these, nearly half required respiratory support, and an attributable case-fatality rate of 5.4% was reported (4). In that study, recipients with recent steroid use and undergoing HSCT within 60 days fared worst from respiratory viral infections. A similar number of associated viral infections were noted in a similar study of SOT recipients, with 14.5% experiencing respiratory viral infections within 12 months but no attributable deaths (4, 5).

**Figure 2:**
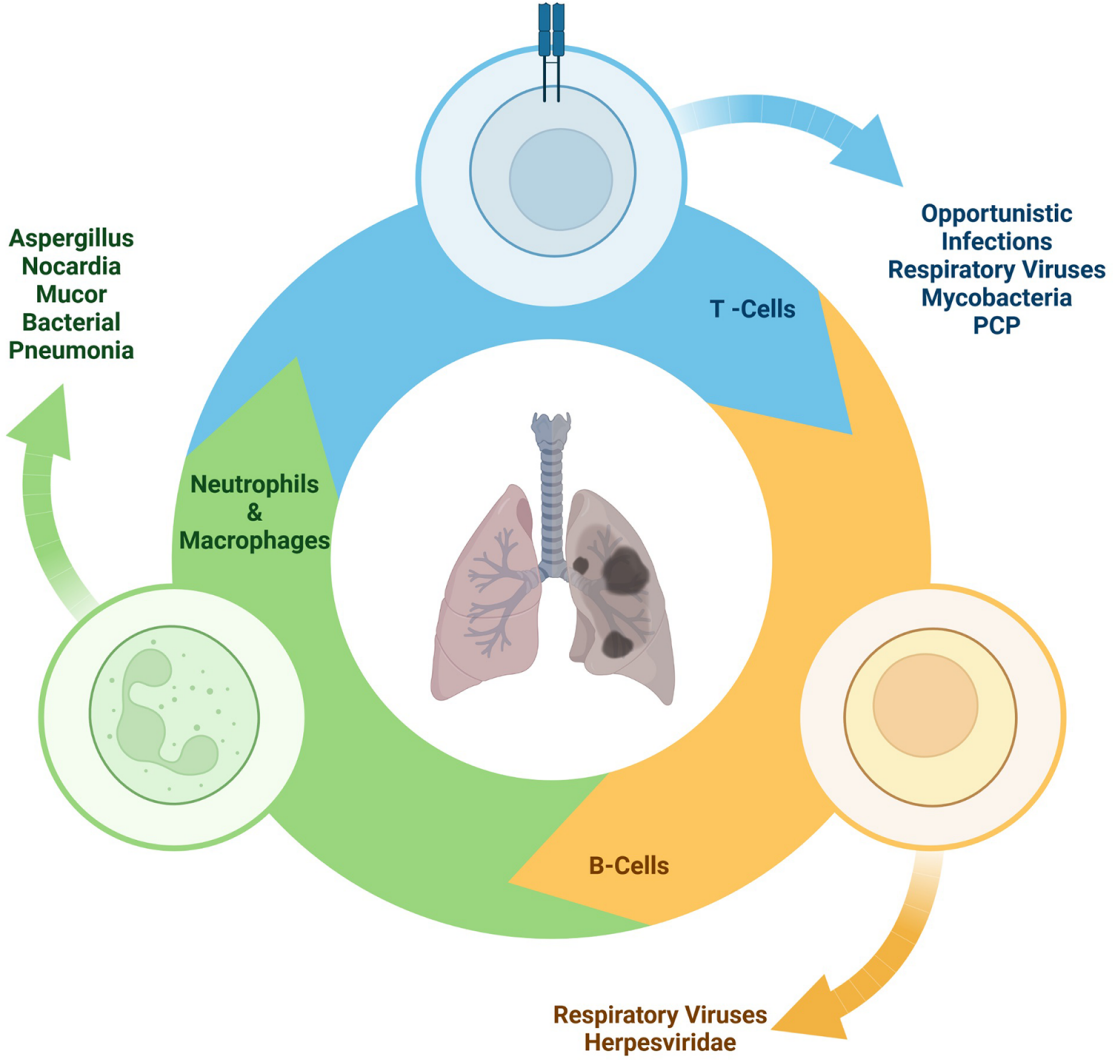
Immune deficits of secondary pulmonary infections.

The incidence of pathogens varies based on many factors, including the type of transplant, the immunosuppression regimen, and the timing post-transplantation. Invasive molds are more prevalent in pediatric HSCT recipients and recipients of lung/heart and lung transplants, with an incidence of around 10%; in contrast, the incidence of invasive molds in other solid organ transplants is closer to 2% (6, 7) (Figure 2).

The pace of clinical evaluation and the depth of the initial differential diagnosis should depend on a combination of clinical stability and estimation of immune function. In a well-appearing transplant patient who is over 1 year out from transplant and on stable immunosuppression, a stepwise approach may be appropriate.

For the presented case, the differential is quite broad, and urgent in-depth evaluation is needed. Her prolonged period of neutropenia elevates her risk of bacterial pneumonia and invasive fungal disease (despite prophylaxis), while her immunosuppression and lymphopenia increase the risk of viral infections and *Pneumocystis* pneumonia. Her recent transplant history of adenoviremia is particularly concerning for viral reactivation with adenovirus, CMV, or the acquisition of a new respiratory virus. Although recent negative weekly screening of blood PCR for adenovirus and CMV is reassuring, the pulmonary disease can occur without concomitant blood DNAemia. PCP pneumonia is a possibility, given her immune status and extended neutropenia, although it is less likely due to the restart of prophylaxis.

For this patient, blood cultures, chest x-rays, and a respiratory panel were immediately requested, and empiric treatment with cefepime was initiated.

**Case**: Chest x-rays showed new bilateral interstitial infiltrates, and a chest CT scan was performed that day. The CT chest scan revealed a new 6-mm ovoid nodule within the anterior segment of the right upper lobe. This nodule was superimposed on new diffuse ground-glass opacities present bilaterally.

***Question 2: What are the potential imaging findings for new pulmonary infections in a transplant recipient?***

In transplant recipients, infiltrates are frequently identified during workup for fever, although the presence of respiratory symptoms is quite variable (8). While chest x-rays are often easier than CT scans to obtain, their interpretation can be difficult and they may be less sensitive.

For acute clinical changes, consolidative lung lesions are commonly associated with bacterial infections. However, subacute consolidations may represent fungal, bacterial, mycobacterial, or *Nocardia* infection. Cavitary disease may be due to bacterial pneumonia (particularly *Staphylococcus aureus* and pneumococcus), mycobacterial disease, or endemic mycoses. Interstitial patterns are commonly associated with viral infections, *Legionella* infections, or *Pneumocystis* pneumonia, but non-infectious etiologies, including pulmonary edema, can also be responsible (9).

CT findings can help arrange a differential diagnosis; however, they can be difficult to interpret, leading to diagnostic uncertainty (10). Even “classic” findings associated with invasive fungal disease (such as a halo or reverse halo sign) are not specific (10). The halo sign represents necrosis and surrounding hemorrhage, which can be due to a number of processes, although it is more frequently seen early in the disease process of invasive mold disease (11). Similarly, pulmonary nodules can be associated with fungal disease or may represent residual inflammation from many potential sources. Nodular disease may also be present with bacterial pneumonia (including *Staphylococcus* and *Pseudomonas*), viral pneumonia (including *Herpesviridae*), *Nocardia*, and mycobacterial infection. Other less specific CT findings that can occur with invasive fungal disease include segmental or peribronchial consolidation with or without tree-in-bud opacities, cavitary lesions with or without air crescent sign, pleural effusions, non-specific ground-glass opacities, and atelectasis (10–12).

**Figure 3:**
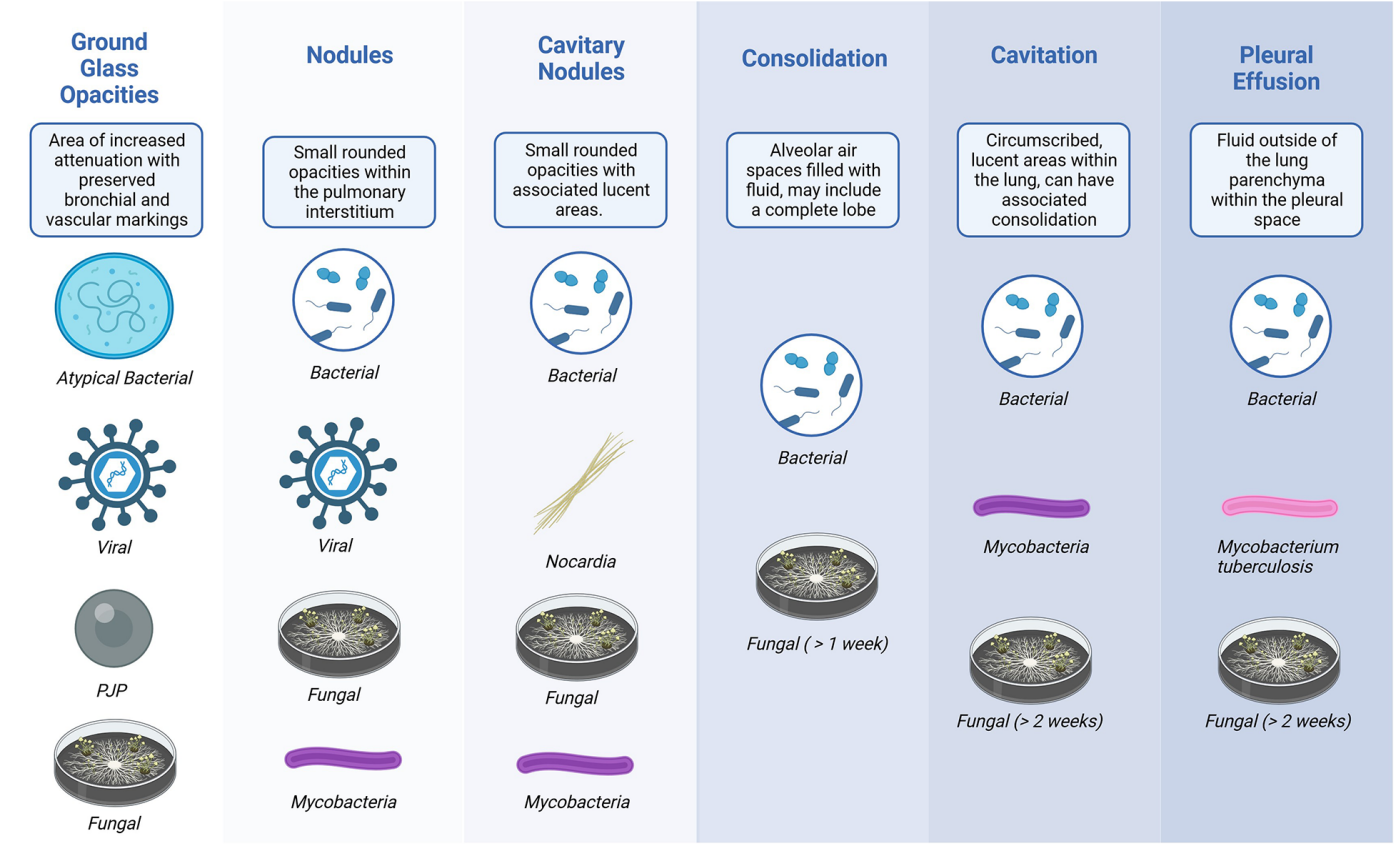
Microbiologic correlation of common CT findings.

It is crucial to recognize that substantial overlap in radiographic findings can be observed, as shown in Figure 3 (13). However, despite this overlap, while complete exclusion may not be feasible, particular imaging findings, as demonstrated in the figure, can help arrange a likely microbiologic differential diagnosis (Figure 3).

For those with neutropenia, detecting fungal infections can be challenging, as classic inflammatory findings may not be present. If initial scans are not conclusive, repeat imaging once neutropenia has resolved may be helpful. However, in high-risk patients, it is generally not advisable to delay scans until neutropenia has subsided (9).

Although not definitive for the presented case, after a review of imaging, a substantial concern for invasive fungal infection was raised. Fungal biomarkers from blood were requested, and an urgent bronchoscopy was scheduled to assist in identifying fungal or other possible infectious etiologies. The existing antifungal therapy (micafungin) was expanded, and liposomal amphotericin was initiated.

**Case**: The blood galactomannan testing returned positive with an *Aspergillus* galactomannan index of 5.7 (normal <0.5), and 1,3-beta-D-glucan was also positive at >500 pg/ml (normal <80 pg/ml); the galactomannan index from bronchoalveolar lavage (BAL) fluid was considered positive at 6.0 (normal <0.5). The patient's respiratory status worsened, and she was unable to be extubated after the bronchoalveolar lavage.

***Question 3: What is the recommended workup for new pulmonary infiltrates in the transplant recipient?***

Once pulmonary imaging is obtained, the evaluation is divided into non-invasive and invasive testing, which are discussed separately. Depending on the concern regarding clinical status and depth of immunosuppression, these tests may be performed consecutively or concurrently.

In stable patients with lower-risk pulmonary findings, such as nodules smaller than 0.5 cm, or well-presenting patients with mild ground-glass opacities/interstitial findings or recent infection that may explain the findings, re-imaging in 2–4 weeks may be reasonable. Non-invasive testing is often performed concomitantly in these scenarios and may inform further workup considerations.

## Non-invasive testing

During the initial evaluation, aerobic blood cultures should be collected. It may be helpful if the patient is able to produce an adequate sputum sample (spontaneous or induced), although it is often not feasible in the pediatric population (14). In newly intubated patients, endotracheal aspirate should be collected if possible (14). Concurrently, all patients should undergo respiratory viral testing with PCR, complemented with serum evaluation where feasible, focusing on CMV and adenovirus. It should be kept in mind that discordant DNAemia between the blood and lungs can occur with CMV.

The mainstay of non-invasive evaluation is fungal biomarkers, particularly 1,3-beta-D-glucan and galactomannan. An elevated 1,3-beta-D-glucan level may indicate invasive infections caused by *Candida* spp, *Aspergillus* spp, *Fusarium* spp, *Coccidiodes*, *Histoplasma*, and *Pneumocystis*, among others*.* 1,3-beta-D-glucan is not detected in infections caused by *Cryptococcus* or *Mucorales*. An elevated galactomannan level may represent infections caused by *Aspergillus* spp, *Penicillium*, *Histoplasma*, or *Cryptococcus*. It is not associated with infections caused by *Candida* or *Mucorales*. There are issues with sensitivity and specificity for both assays, with a particularly high false positivity noted in 1,3-beta-D-glucan (15). Common etiologies of false positives include intravenous immunoglobulin (IVIG), albumin, bacterial sepsis, hemodialysis membranes, surgical gauze, some medications, and some dialysis circuits (15). The false positive etiologies for galactomannan include popsicle sticks, PlasmaLyte, and IVIG, and it is cross-reactive from complex sugars and nutritional supplements (particularly in the setting of mucosal barrier disruption) (16).

The performance characteristics of fungal biomarkers differ based on the type of transplant and are notably better in HSCT than SOT. Estimates on sensitivity and specificity range broadly (33%–94% for 1,3-beta-D-glucan and 61%–95% for galactomannan), highlighting the need to limit use to instances with a high pre-test probability (14, 17). There is limited utility for routine surveillance, and their discriminatory ability may be further impacted by the use of mold-active antifungal prophylaxis (18).

Irrespective of the biomarker results, invasive testing is generally advocated to substantiate the diagnosis. Invasive testing can also provide insights into speciation and susceptibility testing and aid in evaluating co-infections. Elevated 1,3-beta-D-glucan may represent true infection with *Candida*, *Aspergillus*, and other fungal etiologies, but consideration for potential applicable causes of false positives must be given (15). Information may also be used adjunctively; for example, an elevated 1,3-beta-D-glucan combined with a quantitative PCR for *Pneumocystis* can help support the diagnosis of *Pneumocystis* disease from colonization (19). In the setting of negative galactomannan and 1,3-beta-D-glucan testing with a high concern for invasive fungal infection, *Mucorales* infection should be considered. Fungal blood cultures generally provide lower yield, although they have some utility in disseminated histoplasmosis, *Fusarium*, and some other invasive rare molds. However, even in those particular patients, fungal biomarkers are generally more sensitive (20).

The utilization of testing for cell-free DNA through metagenomic next-generation sequencing with commercial assays is quite variable (21). There are several reports of detection of molds and less common pathogens, although performance characteristics are yet to be ascertained. The clinical implications of these assays may be heightened for immunocompromised hosts (22). In general, these evaluations may be most useful when an etiologic organism is highly suspected but cannot be confirmed and diagnostic invasive sampling is not feasible. However, like many diagnostics, the sensitivity of these assays is likely highest prior to or early in the course of effective therapy, and given the substantial cost of the assay, the role of this assay in the routine evaluation of infections remains to be determined.

The use of PCR of blood to detect *Aspergillus* and *Mucorales* is being evaluated with multiple platforms being developed or used with varying performance; however, these cannot be broadly recommended at this time (23).

At the end of 2021, the American Society of Transplantation convened a consensus conference to delineate the utility of advanced diagnostics in solid organ transplants. Numerous knowledge gaps were identified, with challenges persisting around the optimal evaluation and the tangible impacts on clinical outcomes (24).

## Invasive sampling

As detailed above, establishing the etiology of an infiltrate in a transplant patient through non-invasive measures is often challenging. In addition, sampling of sputum is potentially infeasible in pediatric patients (25). Clinicians often find themselves balancing the pressure to avoid invasive procedures vs. the need to establish the diagnosis as quickly and accurately as possible. This challenge is further exacerbated by the possibility that treatments occurring prior to invasive procedures may impair the yield of diagnostics.

Options for invasive sampling that are considered include BAL or direct tissue sampling, either surgically or with interventional radiology.

BAL may lead to changes in clinical management in the range of 50% of cases and in general were very well tolerated (26–28). One study comparing BAL to lung biopsy in pediatric HSCT patients at a single institution found that 40% of the 101 BALs revealed a pathogen, while 94% of the 19 lung biopsies identified an etiology (29). In this report, biopsy identified etiology in six patients with a negative BAL and non-infectious etiology in two patients.

An adult study evaluating bronchoscopy for *Aspergillus* demonstrated the importance of prompt investigation, finding a yield of 35% if performed within the first 2 days, 15% on days 3 and 4, and 2% on day 5 (30).

Broad-range PCR is another tool that can be used to evaluate for bacterial, fungal, and mycobacteria invasive infections. The yield of this test is best for fresh tissue such as biopsy, where organisms are identified by histopathology (31). However, the yield remains limited, with one large pediatric retrospective study finding that broad-range PCR influenced antimicrobial management in only 5% of patients (32).

Results of testing require clinical correlation because positive tests may represent colonization. This is particularly true for *Pneumocystis* and *Aspergillus*, where asymptomatic colonization has been established (33). Detection of viral DNA may represent active infection, asymptomatic reactivation, or prior infection with residual PCR positivity. Similarly, a positive galactomannan from BAL may also represent *Aspergillus* disease or colonization.

## Approach to testing

Unless a specific organism or disease process is strongly evidenced, we recommend a broad evaluation from pulmonary specimens including pathology, culture, fungal culture, AFB culture and stain, modified acid-fast stain, GMS stains, *Legionella* culture, *Pneumocystis* quantitative PCR, galactomannan testing, CMV PCR, and respiratory viral panel, with consideration for *Aspergillus* PCR, *Mucorales* PCR, and *Nocardia* PCR based on imaging and host factors.

**Case:** The differential diagnosis in this case was heavily influenced by CT findings of a pulmonary nodule, coupled with positive indirect assays: galactomannan and 1,3-β-D-glucan. Considerations included *Aspergillus* and other organisms that yield positive galactomannan results (*Fusarium*, *Histoplasma*, *Blastomyces*, *Penicillium*, *Trichophyton*, *Paecilomyces*, *Alternaria*). Viral tests and bacterial cultures were negative; neither next-generation sequencing nor broad-range PCR was sent in this case. A biopsy could not be performed due to progressive clinical instability. Despite the absence of travel to an endemic area, a urine histoplasma antigen was sent and returned negative.

***Question 4: What is the recommended empiric and definitive therapy for new pulmonary infiltrates in the transplant recipient?***

## Treatment

**Figure 4:**
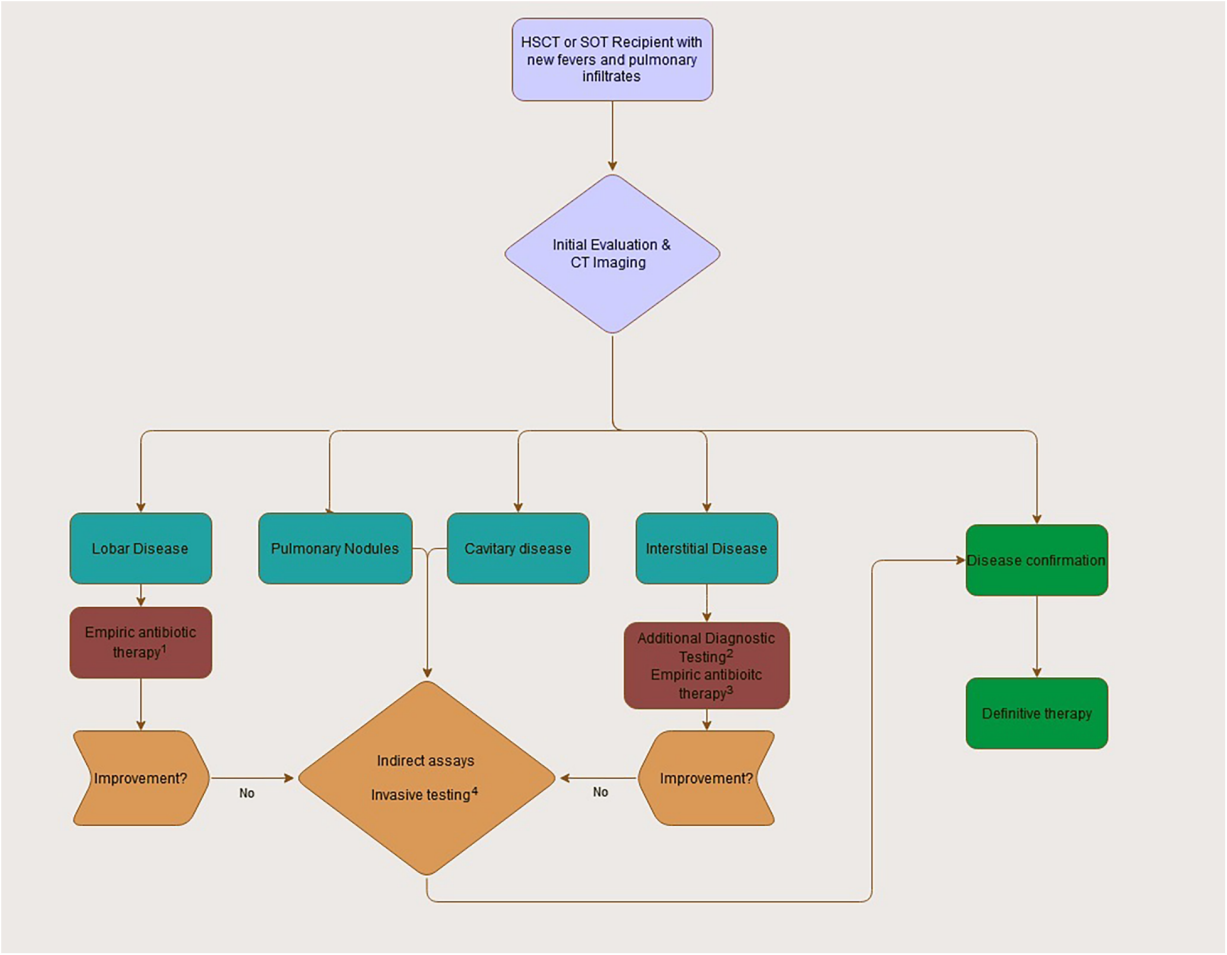
Initial approach to the transplant recipient with fevers and pulmonary infiltrates. (1) Empiric antibiotic therapy will often include a third generation or further cephalosporin with inclusion of pseudomonal coverage when neutropenic. Consideration of additional MRSA coverage should depend on individual staphylococcal risk factors and initial appearance (necrotic pneumonia, severe pleural effusions). (2) Diagnostic assays in interstitial disease can include respiratory viral testing, pneumocystis PCR, and serum viral testing (adenovirus, CMV) as appropriate. (3) Empiric therapy for interstitial disease will depend on severity and presentation at onset given broad differential. Atypical coverage and PJP coverage should be considered when appropriate. (4) Use of invasive sampling (BAL or direct tissue) should be strongly considered for all patients without a clear diagnosis or identified pathogen during initial workup. Empiric antifungal coverage pending workup is prudent.

Considering the broad differential of pulmonary infiltrates in this population, empiric therapy depends on clinical suspicion and is primarily based on history and imaging. Diagnostic evaluation is often unrevealing or returns late into the clinical course, resulting in early treatment decisions made with incomplete information (Figure 4).

Given the frequency and risks of invasive bacterial infection, antibiotics are usually initiated empirically while awaiting additional test results. Empiric antibiotics depend on clinical status and perceived immune status. A beta-lactam with pseudomonal coverage is appropriate in situations where pseudomonal risk is high or the patient is neutropenic, while pseudomonal coverage may not be indicated in patients who have not had recent health system exposure and are more immunocompetent.

For patients with pulmonary nodules at high risk for fungal infection, after obtaining initial labs, empiric treatment is reasonable. The choice of antifungal should be tailored based on clinical presentation, epidemiology, prior antifungal exposures, and available lab results.

In the setting of diagnostic uncertainty, broader coverage, which includes an agent with activity for invasive molds, including *Aspergillus* and *Mucorales*, and others such as posaconazole or liposomal amphotericin, is reasonable. Antifungal selection should consider patient co-morbidities (particularly liver and kidney function), the side-effect profile of agents, and prior prophylaxis (34).

*Aspergillus* is the most common invasive mold, and a regimen providing empiric coverage is important. Medical societies have published guidelines regarding the treatment of pulmonary aspergillosis, and voriconazole is the first-line therapeutic in diagnosed cases (33, 35).

Antifungals with broader coverage than voriconazole are considered alternative therapies in adult *Aspergillus* guidelines. Posaconazole has been found to be non-inferior to voriconazole for primary therapy in adults through day 42 (36). In adults, amphotericin deoxycholate is associated with worse responses and overall survival (37), although a direct comparison with the less nephrotoxic liposomal form has not been done.

For patients developing nodules while on prophylaxis, it may be prudent to empirically expand coverage and prioritize infections not responsive to prophylaxis higher on the differential (presuming medication compliance prior to presentation). Obtaining therapeutic drug concentrations at presentation may help make this decision, and the development of a nodule while on a sub-therapeutic dose may not truly represent drug failure. Azole-resistant *Aspergillus* is a growing concern worldwide, and local epidemiology should be considered in empiric therapy, particularly in the setting of prophylaxis breakthrough. Combination therapy for invasive molds is sometimes utilized, particularly in severe diseases, although data are lacking.

The duration of therapy for fungal infections is highly individualized, and follow-up imaging is required. Concomitant evaluation with invasive diagnostics is strongly recommended in these patients for microbiologic confirmation to guide both the selection and duration of therapeutic choices. This invasive evaluation may also be extended to additional potential secondary sites (sinus, abdomen, etc.) based on presenting symptomology and identified organism. Surgical intervention for *Aspergillus* infections should be considered for severe diseases.

Treatment for viral pneumonia depends on the virus, the immune status of the host, and the severity of the illness. Ribavirin has been used for respiratory syncytial virus, with the greatest benefit observed in those with a history of allogeneic stem cell transplant and lymphopenia (38). In very limited instances, ribavirin has been used for parainfluenza and human metapneumovirus, although data are scant (38). Intravenous ribavirin is not available in the United States. The inhaled formulation is used at some centers, but it poses logistical challenges and is expensive, leading to the increased use of oral ribavirin, if it is used at all.

In high-risk patients, there is a school of thought that antivirals are more likely to be efficacious prior to the progression of lower respiratory tract disease. Numerous respiratory viruses, including adenovirus, influenza, and SARS-CoV-2, have potential directed antiviral therapies available and should be considered when appropriate (38, 39). However, antiviral therapy without confirmation is not recommended. Alternatively, in SOT or HSCT recipients on secondary GVHD-directed immunosuppression with high suspicion or confirmed viral infection, reduction of immunosuppression when feasible may be utilized therapeutically.

CMV should be considered as an etiology of viral pneumonias. Increased DNAemia is correlated with an increased risk of invasive disease, although discordance can occur (40). When CMV disease is suspected or confirmed, consideration of institutional practices regarding primary prophylaxis vs. pre-emptive therapy should inform the need for aggressive evaluation and therapy. The risk for CMV pneumonia is increased for patients without ganciclovir or letermovir prophylaxis, warranting early empiric therapy and diagnostic evaluation. Treatment for mycobacterial infections or *Nocardia* is usually initiated only when additional information supports the diagnosis, given the complexity of selecting an appropriate regimen and the prolonged nature of therapy.

In this case, the patient's condition continued to deteriorate, with growth from the BAL showing 1 + mold, and the patient’s treatment was again expanded to include liposomal amphotericin and posaconazole. Unfortunately, despite antifungal therapy, the patient died from fungal disease. Afterward, the mold was identified as *Scopulariopsis.* A limited autopsy was performed, which confirmed this organism as the cause of death. Cases such as this demonstrate the limitations of the current antifungal arsenal. Hopefully, potential therapeutics in the antifungal pipeline will prove beneficial in similar future situations (41).

## Conclusion

Pulmonary infections in transplant patients are quite challenging to manage as the differential is extremely broad, with the treatment for the different etiologies varying substantially. In general, however, maintaining a rigorous system of evaluation and therapy can help avoid potential pitfalls in this population, where the consequences of misdirected therapy can be substantial. Empiric therapy may be considered for imaging that is largely consistent with specific disease processes. However, in many cases, definitive confirmation is based on a combination of findings from both invasive and non-invasive testing. Whenever feasible, invasive testing should always be pursued and used to guide clinical decision-making. Underuse of bronchoscopy/BAL and lung biopsy likely contributes to significant gaps in potential etiologic identification. Definitive cultures are found in only a minority of cases; however, clinical consideration of indirect assays from both blood and pulmonary specimens can be informative. Therefore, as new modalities such as validated PCRs and next-generation sequencing become increasingly available, they should be rigorously evaluated and incorporated into empiric diagnostic evaluations where reasonable and useful.

## Data Availability

The original contributions presented in the study are included in the article/Supplementary Material; further inquiries can be directed to the corresponding authors.
